# Characterization of aqueous cellulose nanofiber dispersions from microscopy movie data of Brownian particles by trajectory analysis

**DOI:** 10.1039/c8na00214b

**Published:** 2018-10-10

**Authors:** Reiji Motohashi, Itsuo Hanasaki

**Affiliations:** Institute of Engineering, Tokyo University of Agriculture and Technology Naka-cho 2-24-16, Koganei Tokyo 184-8588 Japan hanasaki@cc.tuat.ac.jp

## Abstract

Cellulose nanofibers (CNFs) are promising for various applications such as substrates of flexible devices and reinforcement materials. Most of these applications require control of the drying process of the aqueous CNF dispersions. However, the existing reports examine the surface of dried materials because scanning electron microscopy (SEM) and atomic force microscopy (AFM) are not compatible with either the wet conditions or structure inside the materials. We report the characterization of these aqueous dispersions by the use of optical microscopy although it cannot be used directly to observe CNFs. We add a small portion of colloidal particles into the samples and obtain their trajectory data. The trajectories of Brownian motion include information on the surrounding environments. We analyze the microscopy movie data from the viewpoint of statistical mechanics, and reveal the mesoscale characteristics beyond viscosity. In particular, the possible non-uniformity of the dispersion is quantitatively examined through the framework of the generalized diffusion.

## Abstract 2

Cellulose nanofibers (CNFs) are not directly visible by microscopes when dispersed in water, but the dynamical characteristics of suspended colloidal particles tells us the texture as well as the rheological properties of CNF dispersions beyond the viscosity.

## Graphical abstract

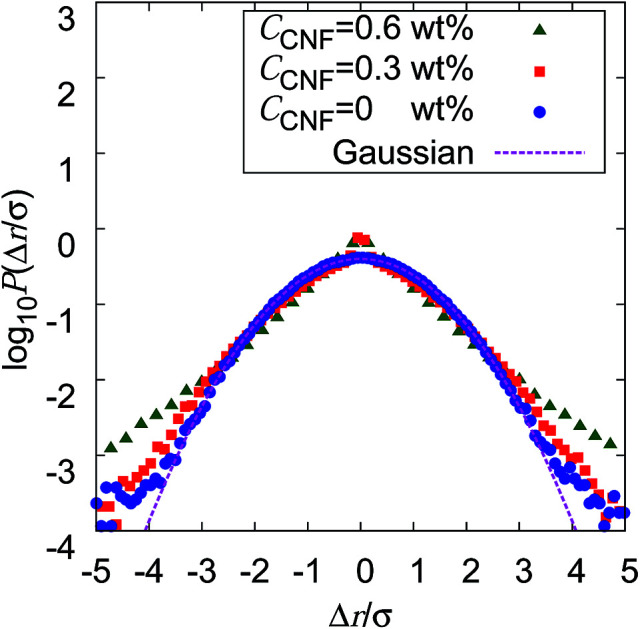

## 1 Introduction

Today, cellulose nanofibers (CNFs)^1^ are well known as environmentally friendly materials, promising for various applications ranging from nanopapers to reinforcements.^2^ Nanopapers are flexible substrate materials made from aqueous dispersions of CNFs by drying them. They are promising for flexible devices in combination with printed electronics^3–12^ because they are not only flexible but also their fine textures are advantageous for keeping the conductive nanoparticles on the surface. The transparent papers can be fabricated from drying the aqueous CNF dispersion,^13,14^ and an electroactive paper actuator made with cellulose/NaOH/urea and sodium alginate^15^ has been reported. Because of their advantage of mechanical strength, CNFs have long been exploited for reinforcement in composites.^16^ The nanocomposites can be highly “green composites” when CNFs are combined with polylactic acid (PLA).^17–20^ Furthermore, mixtures of CNFs with colloidal particles attract attention because of their many potential applications.^21,22^ The nanocomposites where CNFs are filled with colloidal particles have been of interest also in terms of the moisture diffusion in them.^23^ The nanocomposite with CNFs can be fabricated in such a way so as to work as a conductive^24^ or bendable and flexible supercapacitor.^25^ The processing conditions can affect the viscoelastic and electrical properties of nanocomposites filled with CNFs.^26^ Therefore, the rheological behaviors of CNFs have been actively discussed until today.^27–34^

In fact, the drying process to produce these CNF-based nanocomposites is nontrivial. We found in our previous study that the addition of a small amount of CNFs in the colloidal dispersion can suppress the coffee-ring effect of droplets on the substrate surface.^35^ The difference with merely high concentration of particles indicates the special role of CNFs, and implies the existence of the network structure in the last stage of the drying process. Even if the drying is not performed in the state of droplets, the drying temperature and difference of composition can affect the final dried-up state^36,37^ and generally affects the morphology.^33,38,39^ Even the initial concentration of CNFs can affect the transparency of the nanopaper.^12^ Many hydrogen bonds between the filaments of CNFs are formed during the drying of the aqueous dispersion. This stage determines the final structure of CNF-based materials such as nanopapers. Ultrasonication can affect the consequent nanopaper properties.^22^ The blending of different types of CNFs can lead to improved mechanical properties of nanopapers.^40,41^ The network structure of CNFs has been focused on in these existing reports, but it is nontrivial to directly evaluate it unless the samples are dried.^27^

A vast amount of literature indicates the incessant novel findings with respect to the specific properties of CNF-based materials. However, there is an overall feature in the approach to understand the characteristics or mechanism. While the evaluation of functionality is more widespread, the explanation of the mechanism has been less addressed. Existing reports on the texture of CNFs have been mainly based on observation after the drying process by scanning electron microscopy (SEM) or atomic force microscopy (AFM). These approaches are limited to the surface of materials and SEM requires the dried state. The properties of the materials in the wet state or aqueous dispersions have been studied in terms of rheology, where macroscopic evaluation of visco-elastic properties has been carried out. On the one hand, the fine texture of the surface of CNF-based materials after the drying process has been extensively observed. On the other hand, the macroscopic rheology has also been extensively measured. What has been missing in the current state is the link between the states before and after the drying. In other words, the characterization of an aqueous CNF dispersion at the mesoscale between the single filament level and the overall continuum level is desired. This is partly realized by optical coherence tomography (OCT)^42^ and further by X-ray scattering,^43,44^ but other visualization techniques of wet states are based on the macroscopic rheology.^45,46^

In this study, we report a novel approach to reveal the characteristics of an aqueous CNF dispersion. While OCT is mainly used for the flow of the sample and X-rays are used for the static structural properties such as anisotropy, we focus on the characteristics especially when the CNFs are combined with colloidal particles. We suspend the colloidal particles in the samples and obtain the movie data of optical microscopy. Although the CNFs are not visible through the ordinary setup of optical microscopy unless they are labeled with fluorescent dyes, the dispersed particles in the samples can be directly observed. Under the condition that the particles are sufficiently small to exhibit Brownian motion, the diffusion coefficient can be evaluated from the trajectory data. The normal diffusion coefficient is directly related to the effective viscosity through the Stokes–Einstein relation. However, the suspensions of CNFs are not necessarily uniform. The consequent Brownian dynamics is not completely the same as that in bulk uniform liquid. We quantitatively evaluate this characteristic based on the theory of statistical mechanics for the contribution to engineering developments. The Brownian motion of the suspended particles gives us information on the surrounding environment, *i.e.*, the physical state of the CNF dispersion. Furthermore, the particle dynamics itself is also directly important in the development of nanocomposites made from mixtures of CNFs and colloidal particles.

## 2 Methodological details

### 2.1 Generalized diffusion coefficient and finite data

The concept of our methodology in this article is based on the so-called single particle tracking (SPT).^47–50^ Although evaluation beyond diffusion coefficient^51–53^ is possible depending on the amount and quality of the data, we focus on the generalized framework of the diffusion coefficient in this article. This is partly because the consecutively tracked number of steps is relatively small. Nevertheless, a sufficient number of samples are collected to perform the analysis using a sufficient number of particles. The diffusivity of the particle of interest is quantitatively expressed by the diffusion coefficient as follows:1
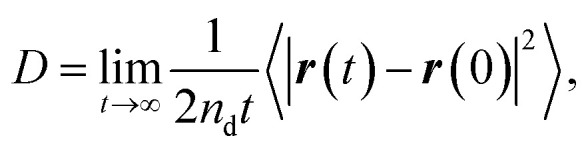
where *n*_d_ = 2 in this system is the dimension of the observation, *r*(*t*) is the position of the particle of interest at time *t*, and 〈⋯〉 indicates the ensemble average. Note that this is consistent with the diffusion equation with the assumption of Fick's law. 〈|*r*(*t*) − *r*(0)|^2^〉 is called the mean squared displacement (MSD). It should also be noted that the relatively small number of consecutive steps originates from the limitation of time resolution by the camera. In other words, the time span to use eqn (1) is easily satisfied for a shortest frame interval of the camera when the system of interest is particles in a liquid.^54,55^ When the particle has spherical shape and the system is sufficiently dilute, the Stokes–Einstein relation predicts the diffusion coefficient *D*_SE_ in bulk as follows:2
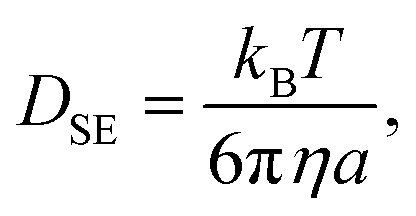
where *k*_B_ is the Boltzmann constant, *T* is the absolute temperature, *η* is the viscosity of the fluid, and *a* is the particle radius. If we measure the diffusion coefficient *D* from eqn (1), eqn (2) provides the corresponding viscosity.

In contrast to the exact definition of eqn (1), the experimentally or numerically available amount of data is always finite. In this case, the possible definition of the diffusion coefficient is not unique. When every frame is regarded to have the equal weight of importance regardless of the individuality of the particle, the following definition can be used:^49^3
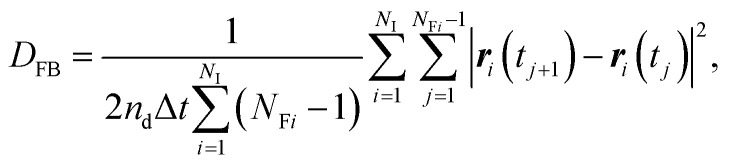
where *N*_I_ is the total number of observed individual particles, *r*_*i*_(*t*_*j*_) is the position of the *i*-th particle at the *j*-th frame, Δ*t* = *t*_*j*+1_ − *t*_*j*_ is the frame interval, *N*_F*i*_ is the number of frames during which the *i*-th particle is consecutively tracked. We call *D*_FB_ the frame-based diffusion coefficient.^49^

On the other hand, when one defines the equality of weight in terms of individual particles instead of each frame, the individual-based diffusion coefficient *D*_IB_ can be used as follows:4
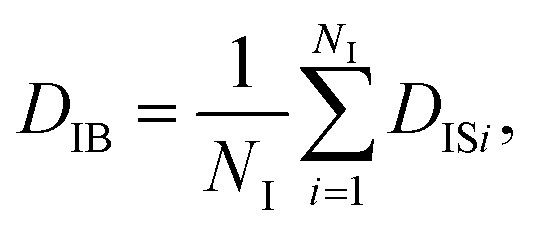
5
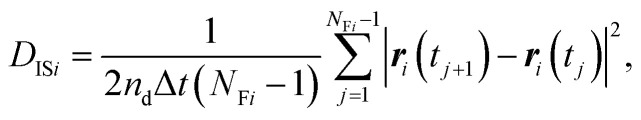
where *D*_IS*i*_ is the diffusion coefficient of the *i*-th particle, and eqn (4) is the ensemble average of *D*_IS*i*_. When all the Brownian dynamics of particles are uniform, eqn (3) and (4) give the same value. In other words, the difference between *D*_FB_ and *D*_IB_ can be used as a hallmark of non-uniformity of the system. Besides the difference of *D*_FB_ and *D*_IB_, the non-uniformity is also evaluated by the following quantity:^56^6
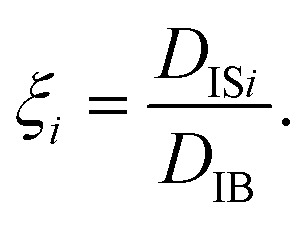


When the particles exhibit normal diffusion, the displacement follows the Gaussian distribution, and the MSD is a linear function of time as implied by eqn (1). However, the Brownian motion of particles in the non-uniform system can exhibit nonlinearity in the time evolution of MSD. Thus, eqn (1) is generalized as follows:^57^

7 〈|***r***(*t*) − ***r***(0)|^2^〉 ≃ *K*_*α*_*t*^*α*^,

and the diffusion coefficient is generalized as8
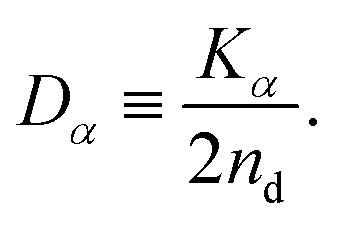


In this article, we call *D*_*α*_ the generalized diffusion coefficient (although *K*_*α*_ is often referred to as such) so that *D*_*α*_ = *D* when *α* = 1. We evaluate the generalized diffusion coefficient *D*_*α*_ and its exponent *α* from the experimentally obtained trajectory data as follows:9
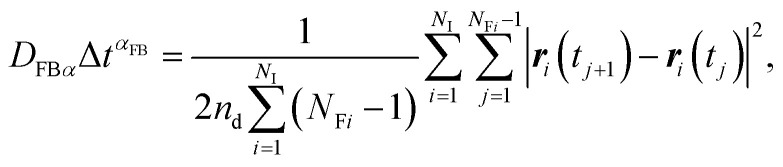
10
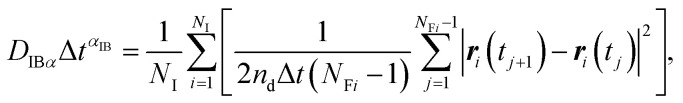
where the subscripts FB and IB indicate the quantities based on the frame-based and individual-based averages, respectively.

As suggested above, the Gaussianity of the displacement distribution is useful for the characterization of the Brownian motion. Besides direct comparison of the distribution *via* histograms, there are statistical quantities to generally characterize the shape of the probability distribution. Kurtosis *K*_u_ is defined as follows:11
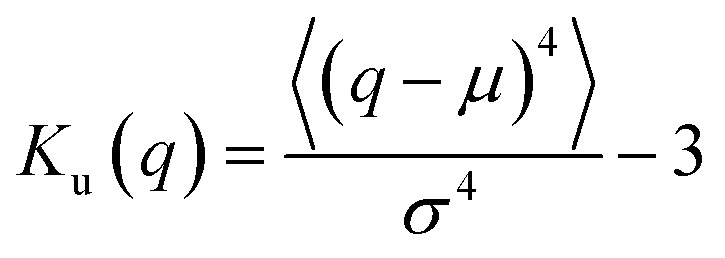
for stochastic variable *q* with mean value *μ* and standard deviation *σ*. *K*_u_ = 0 when the distribution is Gaussian, and *K*_u_ > 0 when it is more concentrated in the vicinity of *q* = *μ*. The asymmetric nature of the distribution is evaluated by the skewness *S*_k_ as follows:12
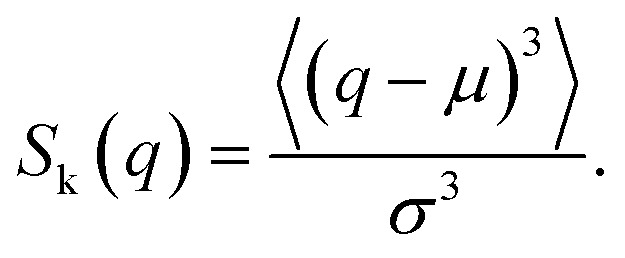


*S*_k_ = 0 when the distribution is Gaussian, and *S*_k_ > 0 when it is biased toward the positive values of *q*. The kurtosis *K*_u_ and the skewness *S*_k_ for the distributions of displacements and *ξ*_*i*_ tell us the characteristics of the stochastic dynamics in the system.

### 2.2 Sample preparation and microscopy measurement

In this study, we employ a type of CNF produced by a mechanical protocol without TEMPO oxidation.^58^ The as-received aqueous CNF dispersion (BiNFi-s, FMa-10002, Sugino Machine, Co., Ltd.) consisted of 2.1 wt% of CNFs. We mixed the CNF dispersion with polystyrene colloidal particles with a diameter of 1.4 μm (Chemisnow, SX-130H, Soken Chemical & Engineering, Co., Ltd.) to prepare the samples with different concentrations of CNFs while keeping the concentration of the particles the same. The overall particle concentration was always 0.1 wt%. The CNF concentration was tuned through dilution with water from 0 to 0.6 wt%. The mixing procedure consists of first shaking the container with a volume of 1.5 mL, second stirring using a handy homogenizer with a pestle (High-power homogenizer, ASG50, AsOne Corp.) at 3000 rpm for 2 minutes. On the other hand, the polystyrene particle dispersion without CNFs was mixed by ultrasonication (UT-106H, Sharp Corp.) at 37 kHz for 20 minutes. We also prepared the CNF/particle mixture dispersion using the ultrasonicator instead of the handy homogenizer to examine the influence of mixing protocols on the uniformity of the sample. The discussion in the next section is based on the sample with the use of the homogenizer unless explicitly addressed.

**Fig. 1:**
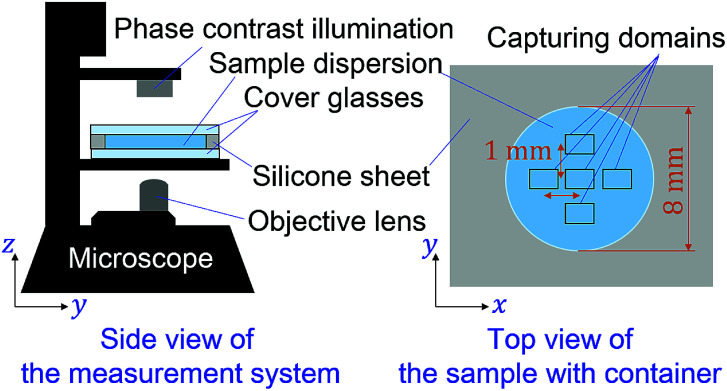
Schematic diagram of the experimental setup.

For each of the concentration conditions, 6 μL of the sample dispersion was placed with a micropipette in containers, with a diameter of 8 mm and depth of 100 μm, which consist of cover glasses on the top and bottom sides with a punched silicone sheet as schematically shown in Fig. 1. The cover glass at the top is meant to avoid evaporation of the water in the samples during observation and possible advection. The sample was placed on the stage for 10 minutes to ensure equilibration before microscopy observation. We obtained the microscopy movie data using an inverted optical microscope (IX73, Olympus, Corp.) and camera (Zyla5.5, Andor Technology, Ltd.). The measurements were conducted for five locations in each sample as shown in Fig. 1. The focus is *ca.* 15 μm away from the bottom cover glass. The pixel pitch of the camera was 6.5 μm, and the magnification of the objective lens was ×20. The captured movie data for each location in a sample consists of 100 frames of time sequential images with a size of 1392 × 1040 px corresponding to 452 × 382 μm. The frame intervals were varied from 0.01 s to 0.4 s to examine the dynamical characteristics.

### 2.3 Particle tracking and trajectory analysis

The particle trajectory data were extracted from the microscopy movie data, using the algorithm of ref. 59. The algorithm requires the input parameter to conduct the tracking. We used the set of parameters summarized in Table 1. Only a single parameter to define the threshold value of possible displacement per frame interval was varied based on the frame interval. More specifically, this threshold value for displacement is determined by the definition of the diffusion coefficient in terms of the mean squared displacement (eqn (1)) and the Stokes–Einstein relation (eqn (2)). When the frame interval Δ*t* is decided and the bulk diffusion coefficient without CNFs is theoretically predicted, the typical displacement per frame is determined. Since the normal diffusion shows a Gaussian distribution of displacements, four times of the typical displacements covers most of the possible displacements and the error is far below other factors. The consequently determined threshold values of possible displacements are summarized in Table 2. In order to discuss the time evolution of mean squared displacements (MSDs), we evaluated the MSDs with different time spans Δ*t*. Δ*t* corresponds to the frame interval in this situation. We captured the movie data under four conditions of frame interval, and we also examined the doubles of these intervals by taking every two frames. We evaluated statistical quantities using 50 frames of the images for every condition of the frame interval.

**Table 1:** Input parameters for the particle tracking to obtain trajectories by the algorithm of ref. 59. It should be noted that “Particle radius” is the size of the particle image. “Intensity Percentile” stands for the threshold value of the light intensity to regard the spot as the particle of interest. “Cutoff score” defines the threshold value by the distinction of light intensity distribution characteristics, and 0 means that there is no distinction. “Link range = 1” indicates that only directly consecutive frames were analyzed for the particle identification to get displacements

Parameter	Value
Particle radius (pixel)	4
Intensity percentile (%)	0.3
Cutoff score (—)	0
Link range (frames)	1

**Table 2:** The threshold value of possible displacements for the particle tracking analysis to obtain trajectories by the algorithm of ref. 59. The rest of the parameters are summarized in Table 1

Frame interval (s)	Displacement threshold (pixel)
0.01	2
0.02	3
0.03	3
0.05	4
0.06	4
0.1	5
0.2	7
0.4	10

## 3 Results and discussion

**Fig. 2:**
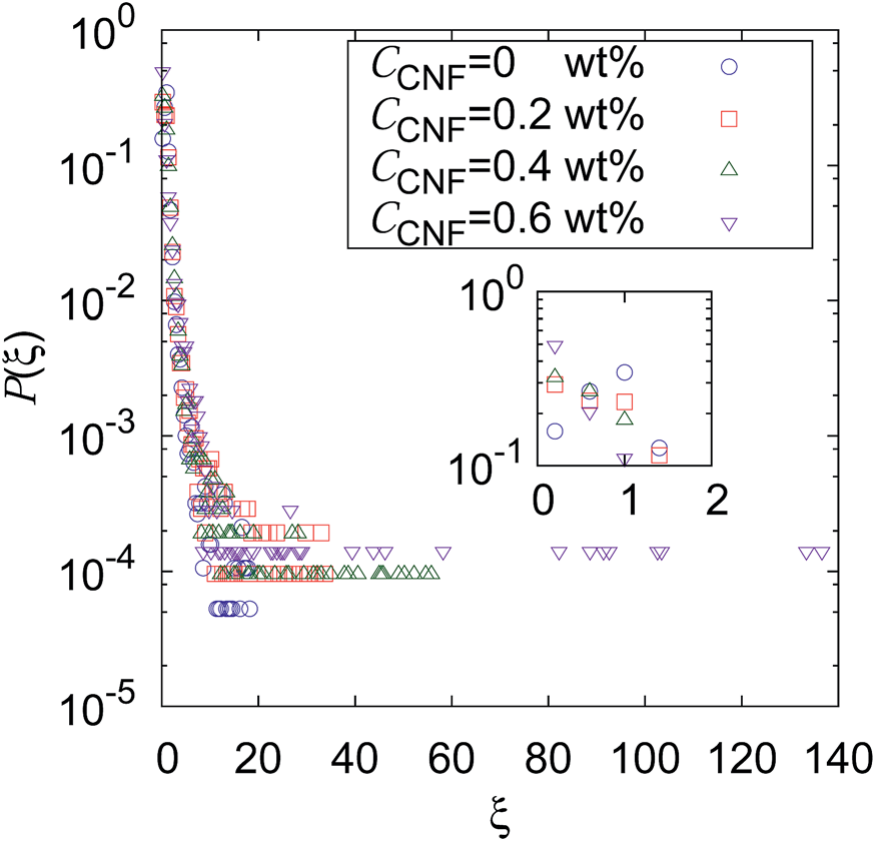
Distribution of individual-based diffusion coefficients expressed as *ξ* defined in eqn (6) when the time span is 0.4 s for different CNF concentrations. The inset shows the zoomed in figure around *ξ* = 1, *P*(*ξ*) = 0.5.

**Fig. 3:**
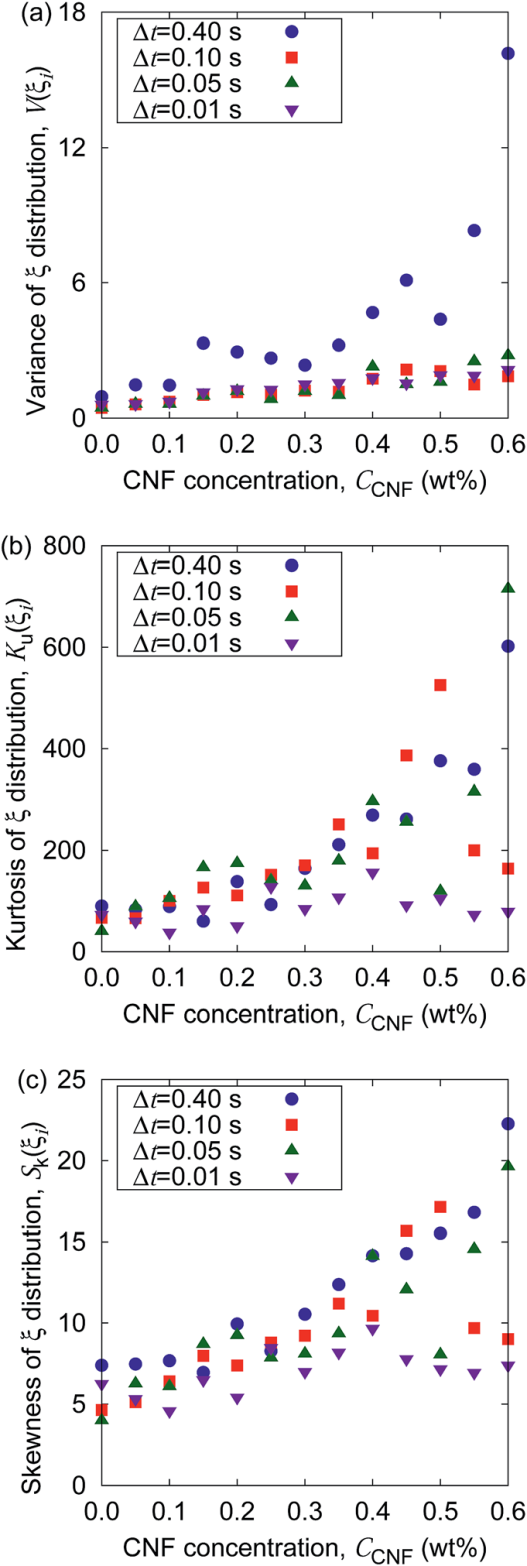
Dependence of the shape characteristics of *ξ* distribution on CNF concentration: (a) variance, (b) kurtosis, and (c) skewness.

Fig. 2 shows the distribution of *ξ* defined in eqn (6). It can be seen from this figure that there are samples with larger *ξ* for higher CNF concentrations. Further characterization of this distribution is summarized in Fig. 3. The variance, kurtosis, and skewness of *P*(*ξ*) increase with the increase of CNF concentration. The increase of variance indicates the overall broadening of the distribution. On the other hand, the increase of kurtosis indicates a sharper peak around the expected (or mean) values. Apparently these features contrast with each other. However, the distributions of *ξ* for higher CNF concentrations have higher probabilities for both small and large *D*_IB_, while having lower probability for the intermediate values. In addition, the larger skewness of *ξ* distribution indicates an increase in the larger *ξ* compared to the smaller ones, which is also directly read from Fig. 2 as just mentioned above.

**Fig. 4:**
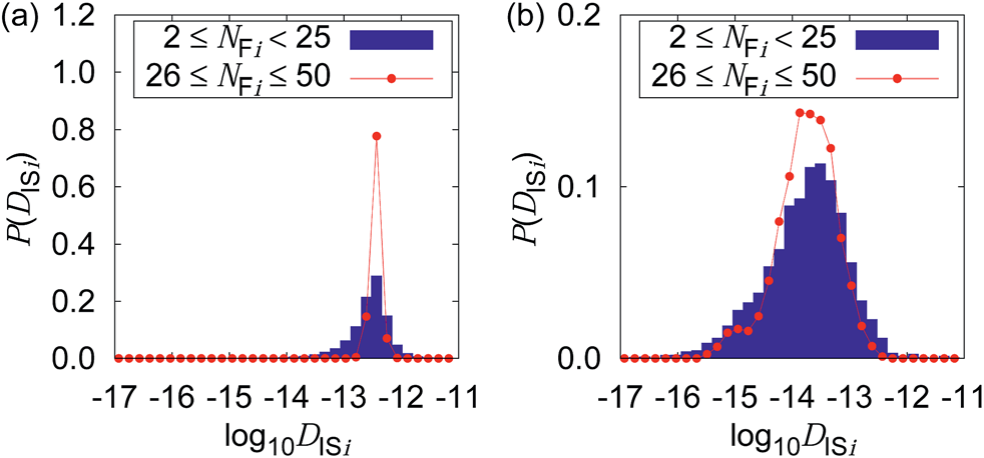
Distribution of the logarithms of the diffusion coefficient for Δ*t* = 0.4 s when the CNF concentrations are (a) 0 wt% and (b) 0.6 wt%, respectively. The dependence of the consecutively tracked duration (corresponding to *N*_F*i*_) is plotted for each figure.

Fig. 4 shows the possible dependence of *D*_IS*i*_ distribution on the consecutively tracked duration, *i.e.*, *N*_F*i*_. While the bulk water shows the independence of *D*_IS*i*_ from *N*_F*i*_, the mixture with CNFs at 0.6 wt% shows the lower peak of log_10_ *D*_IS*i*_ for longer tracked durations (*i.e.*, larger *N*_F*i*_). When the dynamics of individual particles are uniform as shown in Fig. 4(a), the difference in *N*_F*i*_ results only in the breadth of the distribution. This is a consequence of the central limit theorem. The difference in the most typical *D*_IS*i*_ correlated with *N*_F*i*_ suggests a kind of non-uniformity of the Brownian dynamics in terms of the timescale. Therefore, we are going to examine it by the framework of the generalized diffusion coefficient.

**Fig. 5:**
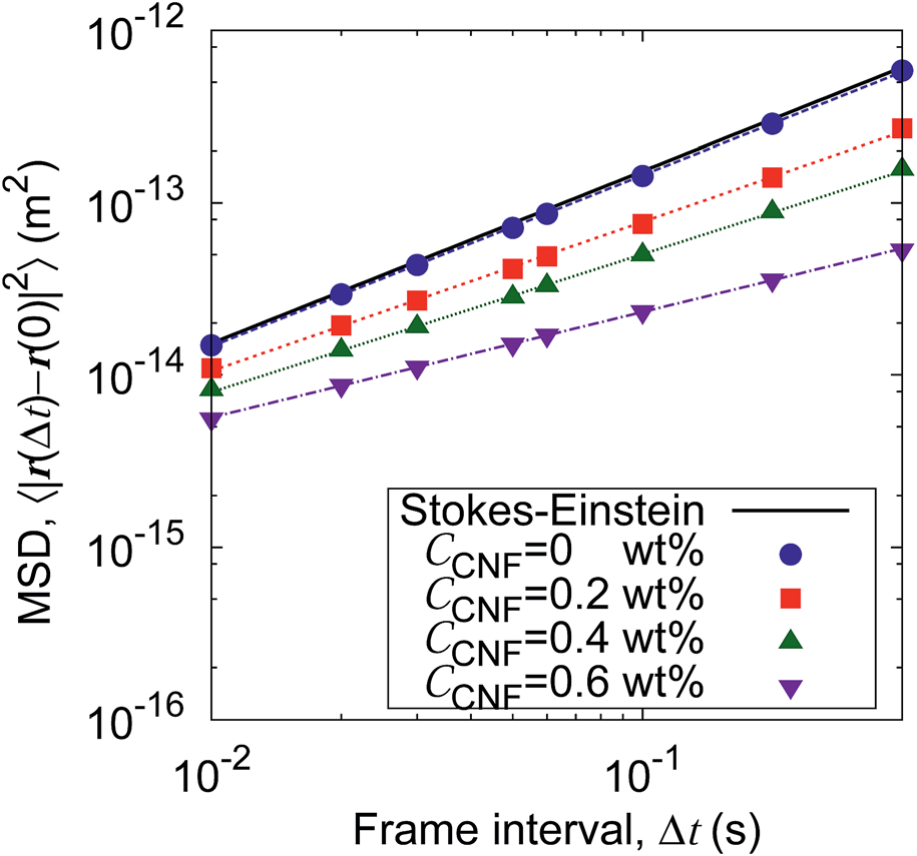
Time evolution of the mean squared displacements (MSDs) for different concentrations of CNFs. The solid line indicates the prediction by the Stokes–Einstein relation.

Fig. 5 shows the time evolution of the MSDs for different CNF concentrations. The average temperature in the experiments was 28.4 °C, and hence the diffusion coefficient for bulk particle dispersion without CNFs was estimated using the Stokes–Einstein relation *D*_SE_ = 3.8 × 10^−13^ m^2^ s^−1^. The experimental result of the MSD as a function of time for particle dispersion without CNFs agrees with this value. On the other hand, the MSDs are smaller for higher CNF concentrations. The slope of this double logarithmic plot is also smaller for higher CNF concentrations. Thus, the existence of CNFs in the dispersion slows down the diffusion of suspended particles.

**Fig. 6:**
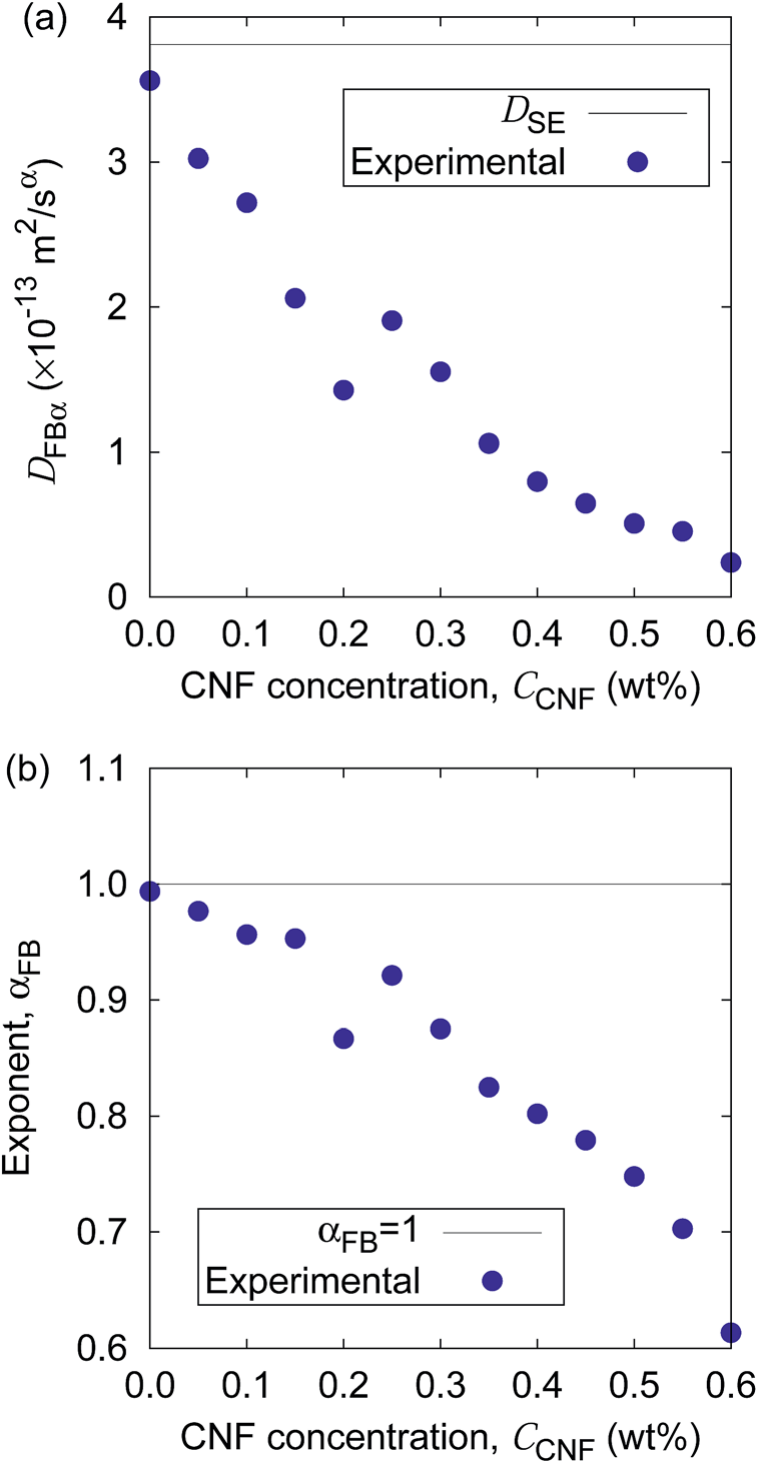
The CNF concentration dependence of (a) the generalized diffusion coefficient *D*_*α*_ and (b) its exponent *α*. The solid lines indicate the diffusion in bulk water (*i.e.*, *C*_CNF_ = 0) predicted by the Stokes–Einstein relation.

Furthermore, the apparent linearity in the double logarithmic plot suggests the validity of the framework of the generalized diffusion coefficient. Therefore, we extract the coefficients and exponents of the generalized diffusion coefficients as shown in Fig. 6. From this set of figures, it can be confirmed again that the Brownian motion without the CNFs exhibits the normal diffusion, *i.e.*, *α* = 1, and the diffusion coefficient matches the prediction by the Stokes–Einstein relation as already mentioned. It is now also clear that both the coefficient *D*_*α*_ and exponent *α* of the generalized diffusion decrease with increasing CNF concentration. The decrease of *D*_*α*_ and *α* is not a sudden drop with a threshold but gradual with the CNF concentration. In other words, the variation of the physical state within this range of concentrations is less likely to be the typical phase transition.

**Fig. 7:**
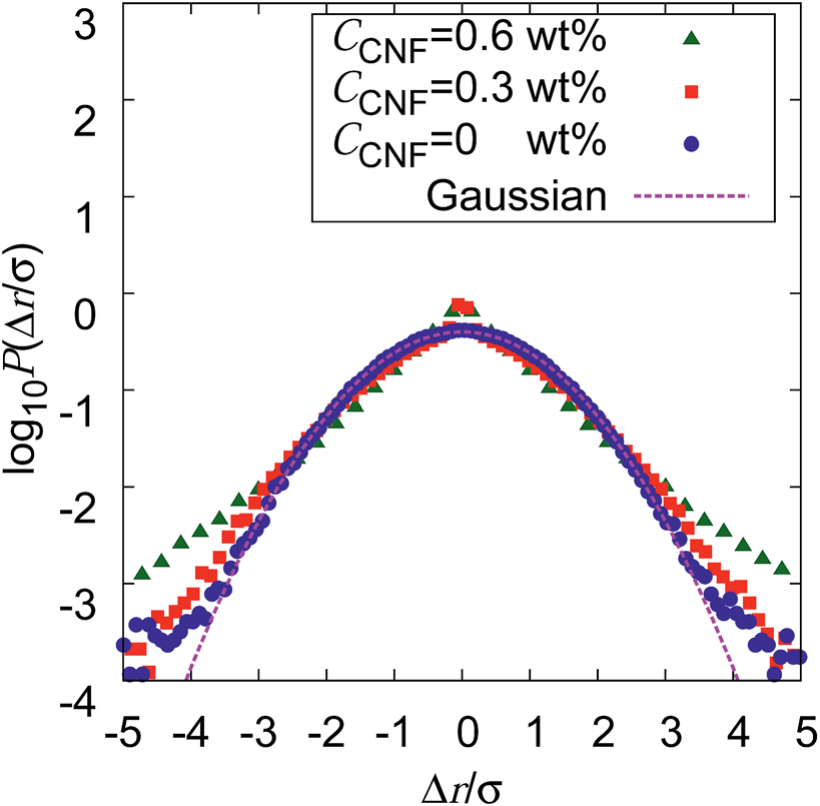
Displacement distribution for Δ*t* = 0.4 s when the CNF concentrations are *C*_CNF_ = 0, 0.3, and 0.6 wt%. The displacement Δ*r* stand for the equivalent *x* and *y* components of the two dimensional displacements. Δ*r* is scaled by the standard deviation *σ*. The probability *P*(Δ*r*/*σ*) is normalized in such a way that the integrated area becomes 1, in order to compare the shape of the probability distribution with the Gaussian.

If the slowing down of the diffusion is purely caused by the viscosity of the fluid, *α* should remain at 1 with the decrease of *D*_*α*_. The significant decrease of *α* indicates that the nature of Brownian motion is affected by the CNFs. This characteristic of Brownian motion is also recorded in the displacement distribution as shown in Fig. 7. This figure clearly shows that the non-Gaussian distribution emerges and is manifested as the CNFs are added to the samples and the concentration is increased. While the particle dispersion without CNFs shows a roughly Gaussian distribution, the mixtures with CNFs exhibit distributions with higher probability for the smallest displacements and sufficiently large ones.

The decrease of *α* below 1 directly indicates the confinement effect of Brownian motion, and the non-Gaussian displacement distribution suggests the hindrance to particle diffusion by the fiber structures. Thus, the Brownian particle trajectory analysis reveals the mesoscopic physical state of the CNF dispersion, which is not directly accessible using SEM, *etc.* The decrease of *D*_*α*_ is partly attributed to the effective increase of the viscosity by the existence of dispersed materials and the existence of a solid structure in the vicinity of the Brownian particles. This is because the existence of a solid wall generally causes the slowing down of Brownian motion in the close vicinity by the hydrodynamic effect.^60–66^

**Fig. 8:**
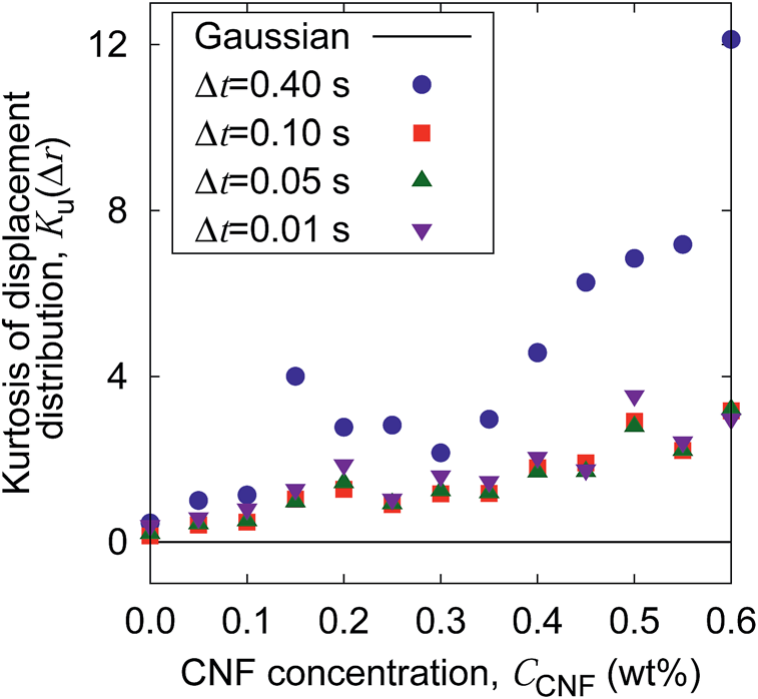
The CNF concentration dependence of the kurtosis for the displacement distribution. The displacement distribution corresponds to Fig. 7. The solid line *K*_u_(Δ*r*) = 0 corresponds to the normal diffusion.

The increase of probability around zero displacement is quantitatively extracted as the kurtosis for all concentration conditions in Fig. 8. It can be due to two possible reasons. One is that the increase of probability for the smaller displacements is caused by the constraint of the Brownian dynamics of suspended particles with the structures of CNFs and intermittent large displacements take place when the particles overcome this spatial hindrance by thermal fluctuation. The other is that the non-Gaussian displacement distribution is caused by the non-uniformity of the surrounding medium beyond the range of the expected pore size formed by the uniform CNF distribution with random orientations.

**Fig. 9:**
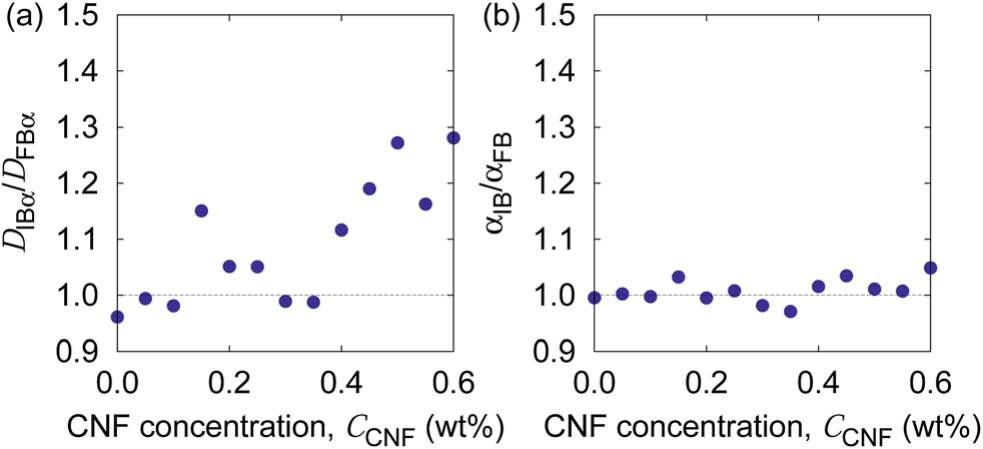
The ratio of individual-based to frame-based diffusion coefficients as a function of the CNF concentration.

In order to examine these two possibilities that can simultaneously take place, we show in Fig. 9 the ratio of individual-based to frame-based values of the generalized diffusion coefficients and their exponents. The generalized diffusion coefficient is larger for the individual-based values compared to the frame-based ones, *i.e.*, *D*_IB*α*_/*D*_FB*α*_ > 1, and appears to have a positive correlation with the CNF concentration. This indicates that the individual particles with larger *N*_F*i*_ tend to have smaller diffusion coefficients, or the particles with smaller *N*_F*i*_ tend to have larger diffusion coefficients. On the other hand, the scaling behavior of MSD, *i.e.*, the exponent for the time dependence, is robust against this kind of non-uniformity, *i.e.*, *α*_IB_/*α*_FB_ = 1. This implies that the spatial confinement effect by the CNFs is sufficiently uniform based on the observation timescale of 0.4 s. In other words, the particles tend to experience both confinement effect and relatively free diffusion during the timescale of *O*(10^−1^) s. Nevertheless, the clear trend of *D*_IB*α*_/*D*_FB*α*_ > 1 and its enhancement by the higher CNF concentration indicates the variation of texture formed by the CNFs. While it remains to be concluded, it is likely that the smaller pore size distribution by the CNF dispersion causes the enhanced effect of hydrodynamic slowdown of the Brownian motion of particles. The hydrodynamic slowdown effect is manifested only when the particle is in the vicinity of the solid structure within the distance in the order of the particle diameter.^61,62^ This point is currently beyond the scope of this study.

**Fig. 10:**
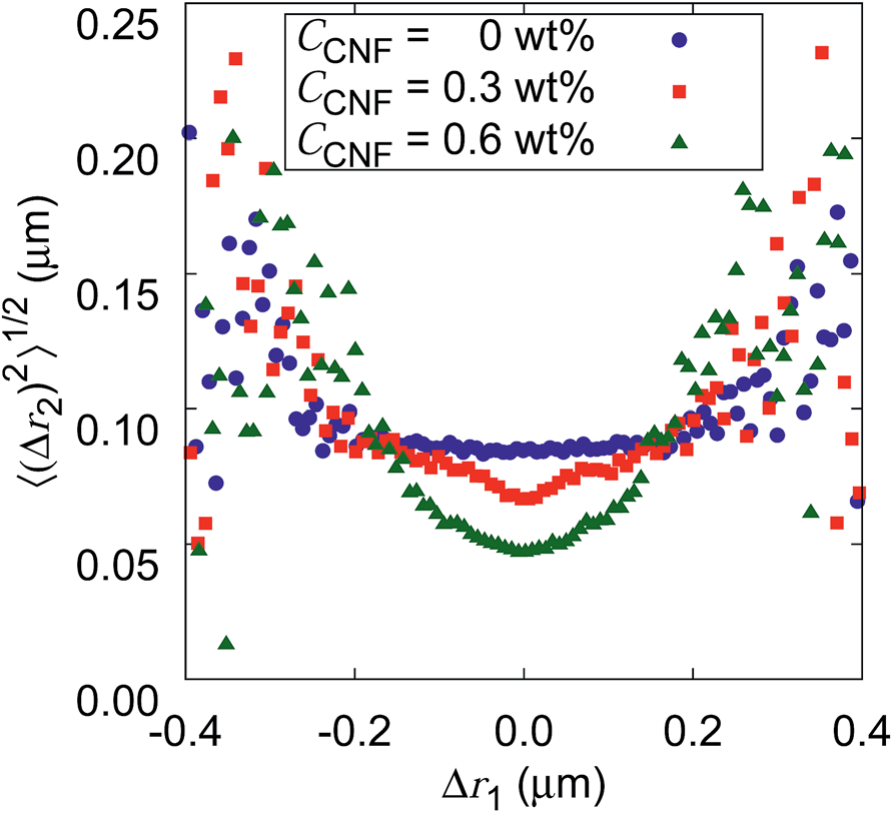
The relation between the consecutive displacements for different CNF concentrations. Δ*r*_2_ is the displacement just after the Δ*r*_1_ when the frame interval Δ*t* = 0.01 s. Δ*r*_1_ is binned and corresponding 〈(Δ*r*_2_)^2^〉^1/2^ are plotted as the histograms.

Fig. 10 shows the root mean squared displacements (root MSDs) as a function of the previous displacements. This plot represents a kind of time correlation for a pair of successive displacements.^56^ The Brownian motion of the particles in water without CNFs shows no time correlation. As a result, the plot takes the form of a flat line. The large scatter on both ends of the large displacements is simply caused by the poor number of samples regardless of the CNF concentration condition. When the CNFs are added to the particle dispersion, the plot has a concave shape. This indicates that small displacements tend to follow small displacements. Furthermore, the value of root MSDs after the same length of the previous displacements becomes smaller for higher CNF concentrations. This is because of the more significant confinement effect for higher CNF concentrations. Since the addition of CNFs to the system only slows down the diffusion, root MSD after arbitrary Δ*r*_1_ does not exceed the value for the pure particle dispersion without CNFs.

If the CNF dispersion consists of a highly non-uniform structure with large voids without CNFs at the space scale where the particles can travel within a timescale of 10^−1^ s, this plot would exhibit a horizontally flat shape for large displacements within the typical scale for the diffusion in bulk water. Therefore, the non-Gaussian displacement distribution (*cf.*Fig. 7) with a subdiffusive scaling exponent (*i.e.*, *α* < 1) originates from the continuous traveling of the particles between more confined subdomains and less confined ones without significantly isolated voids to trap the particles. The particle dynamics is less of the trap and jump, and more of the continuous variation. This is consistent with the discussion based on Fig. 9.

**Fig. 11:**
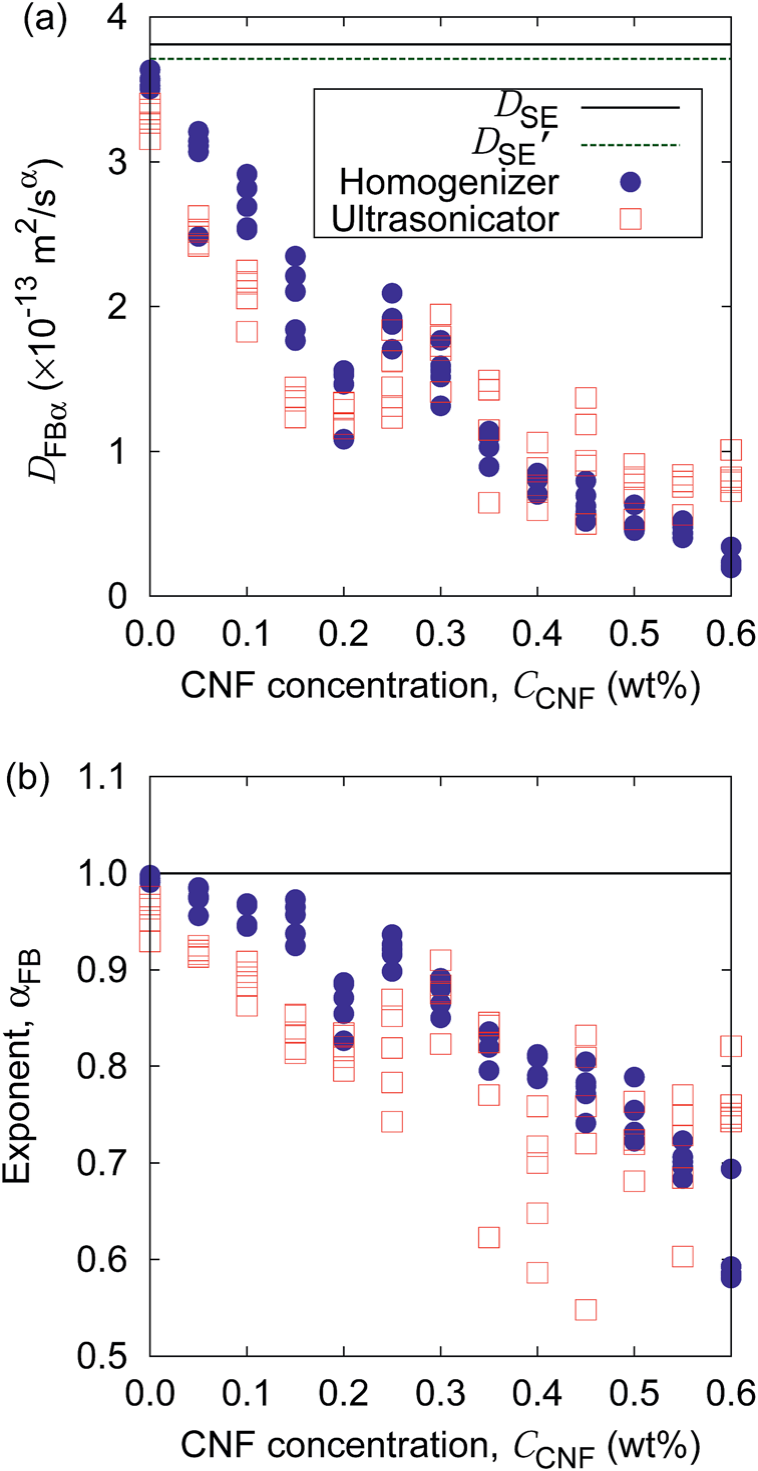
Comparison of mixing protocols to prepare the aqueous CNF dispersions by (a) the generalized diffusion coefficient and (b) its exponent as a function of CNF concentration, plotted as the scatter diagrams. The lines indicate the prediction by the Stokes–Einstein relation. *D*_SE_ and *D*′_SE_ indicate the normal diffusion coefficients at the temperatures when the handy homogenizer or ultrasonicator is used, respectively.

Finally, we demonstrate the evaluation of the possible non-uniformity of the samples caused by the difference of mixing protocols. Fig. 11 shows the generalized diffusion coefficients and their exponents obtained at five locations of each sample (*cf.*Fig. 1) at specific CNF concentrations prepared either by the mixing with a handy homogenizer or a normal ultrasonicator. Although the difference of the coefficients is not clear, the exponents of the generalized diffusion coefficient show a difference in the scatter plots. The breadth of the *α*_FB_ distribution for each *C*_CNF_ tends to be larger for the case of the ultrasonicator alone when *C*_CNF_ > 0.2 wt%. The samples prepared without the homogenizer tend to show significant non-homogeneity at a space scale of 1 mm (*cf.*Fig. 1) when the CNF concentrations are sufficiently high. It should be noted that we employed only a single type of homogenizer and ultrasonicator, respectively. There are numerous differences in the specification of the devices for mixing, and we do not intend to explain the general trend between the homogenizer and the ultrasonicator in this article. Instead, we intend to demonstrate the usefulness of this particle tracking analysis for the quantitative evaluation of the diverse possibilities of processing protocols before the drying.

## 4 Conclusions

We have presented the application of statistical mechanics for the characterization of an aqueous CNF dispersion through Brownian particle dynamics. The detailed evaluation of the Brownian dynamics of suspended particles in the CNF dispersion helps us understand the structures of CNFs in water and the diffusion of particles themselves. The former is always important in the production of CNF-based materials from the aqueous dispersion. The latter is also important when CNFs are combined with colloidal particles to develop composites for which the functionality is realized by the nanoscale structural characteristics. We have demonstrated only a set of symbolic case studies to explain the usefulness of this approach. The diversity in the difference of CNFs themselves, processing conditions, and particles implies that there is plenty of room at the mesoscale for exploration. The exploration will link the SEM observation in the dry state and rheological measurements in the wet state, where the former is microscopic and the latter is macroscopic in many of the existing reports.

## Conflicts of interest

There are no conflicts to declare.

## Supplementary Material
